# A reflection on the use of Antidepressants to manage agitation in dementia

**DOI:** 10.1192/j.eurpsy.2022.1683

**Published:** 2022-09-01

**Authors:** M.D.R.D.R.F.D. Basto, L. Santa Marinha, O. Nombora, A. Horta

**Affiliations:** Centro Hospitalar de Vila Nova de Gaia e Espinho, Serviço De Psiquiatria E Saúde Mental, Vila Nova de Gaia, Portugal

**Keywords:** agitation, Antidepressants, Dementia

## Abstract

**Introduction:**

Agitated behaviors is a common neuropsychiatric symptom (NPS) in dementia, defined as inappropriate verbal, vocal, or motor activity that is not thought to be caused by an unmet need. It is frequently reported as a major problem, that impairs the quality of life for the elderly themselves and for caregivers. There has been increasing interest in the use of sedative antidepressants to treat NPS due to concerns over the safety and efficacy of antipsychotics in this setting.

**Objectives:**

We aim to review clinical evidence of alternatives to antipsychoticst to manage agitation in dementia.

**Methods:**

We conduct a non-systematic review of recent evidence on dementia and agitation, using PubMed/Medline database.

**Results:**

Although non-pharmacological interventions are the first-line treatment for agitation, it is a legitimate target for therapeutic intervention and according to previous guidelines, antipsychotic are among the most used drugs, albeit restricted because of side-effects. A substitution strategy to avoid antipsychotic prescription was highly considered, however there is limited evidence to support the use of antidepressants as a safe and effective alternative for agitation in dementia. Studies compare Mirtazapine, Selective serotonin reuptake inhibitors (SSRIs) and Trazodone and a reduced benefit in mortality is observed. However, citalopram was more effective were more likely outpatients for moderately agitation and Mirtazapine reveals being potentially harmful, in different studies.

**Conclusions:**

Moving forward, a greater understanding of NPS neurobiology, will help to clarify the efficacy of Antedepressants for the treatment of agitation in dementia. Benefits an also the patient and caregiver preference should be kept in mind.

**Disclosure:**

No significant relationships.

